# Serotype Distribution and Disease Severity in Adults Hospitalized with *Streptococcus pneumoniae* Infection, Bristol and Bath, UK, 2006‒2022

**DOI:** 10.3201/eid2910.230519

**Published:** 2023-10

**Authors:** Catherine Hyams, Robert Challen, David Hettle, Zahin Amin-Chowdhury, Charli Grimes, Gabriella Ruffino, Rauri Conway, Robyn Heath, Paul North, Adam Malin, Nick A. Maskell, Philip Williams, O. Martin Williams, Shamez N. Ladhani, Leon Danon, Adam Finn

**Affiliations:** University of Bristol, Bristol, UK (C. Hyams, R. Challen, R. Heath, L. Danon, A. Finn);; Southmead Hospital, Bristol (C. Hyams, C. Grimes, G. Ruffino, R. Conway, N.A. Maskell, A. Finn);; Bristol Royal Infirmary, Bristol (D. Hettle, P. North, P. Williams, O.M. Williams);; UK Health Security Agency, London, UK (Z. Amin-Chowdhury, S.N. Ladhani);; The Royal United Hospital, Bath, UK (A. Malin)

**Keywords:** pneumonia, Streptococcus pneumoniae, bacteria, pneumococcus, serotypes, serotype distribution, bacterial infections, disease severity, respiratory infections, hospitalized adults, Bristol, Bath, United Kingdom

## Abstract

Ongoing surveillance after pneumococcal conjugate vaccination (PCV) deployment is essential to inform policy decisions and monitor serotype replacement. We report serotype and disease severity trends in 3,719 adults hospitalized for pneumococcal disease in Bristol and Bath, United Kingdom, during 2006–2022. Of those cases, 1,686 were invasive pneumococcal disease (IPD); 1,501 (89.0%) had a known serotype. IPD decreased during the early COVID-19 pandemic but during 2022 gradually returned to prepandemic levels. Disease severity changed throughout this period: CURB65 severity scores and inpatient deaths decreased and ICU admissions increased. PCV7 and PCV13 serotype IPD decreased from 2006–2009 to 2021–2022. However, residual PCV13 serotype IPD remained, representing 21.7% of 2021–2022 cases, indicating that major adult PCV serotype disease still occurs despite 17 years of pediatric PCV use. Percentages of serotype 3 and 8 IPD increased, and 19F and 19A reemerged. In 2020–2022, a total of 68.2% IPD cases were potentially covered by PCV20.

## Abstract 2

Pneumococcal conjugate vaccinations should be evaluated and considered in formulating future public health policy recommendations.

*Streptococcus pneumoniae* remains the leading bacterial cause of community-acquired pneumonia, despite widespread use of effective pneumococcal vaccines with >100 recognized pneumococcal serotypes. In the United Kingdom, unconjugated 23-valent pneumococcal polysaccharide vaccine (PPV23) is offered to all adults >65 years of age and persons >2 years of age who are at increased risk for pneumococcal disease. In September 2006, a 7-valent pneumococcal conjugate vaccine (PCV7) was implemented into the national childhood immunization program, then replaced with a 13-valent PCV (PCV13) in April 2010. The PCVs were given at a 2 + 1 schedule but replaced with a 1 + 1 schedule in April 2020 (*1*). Because PCVs prevent carriage acquisition in addition to protection against disease, both PCVs had a large and major direct and indirect (herd) effect on pneumococcal disease caused by the respective serotypes (*2*–*4*).

By 2016‒2017, PCV7 pneumococcal serotype disease had virtually disappeared in children and decreased to a large degree in adults. Invasive pneumococcal disease (IPD) caused by PCV13 serotypes has also decreased substantially but subsequently plateaued, with a residual incidence of 8 IPD cases/100,000 population in England (*5*–*7*). At the same time, pneumococcal cases caused by non-PCV13 serotypes increased across all age groups, especially in older adults, resulting in no net reduction in total IPD cases in older adults in 2016‒2017 compared with the pre-PCV13 period, a phenomenon known as serotype replacement (*5*–*7*). Monitoring those effects nationally is vital for planning of healthcare use and developing new preventive strategies, including use of higher-valent pneumococcal vaccines (*8*,*9*).

The effect of serotype replacement has not been reported in the United States; data suggest that the incidence on nonvaccine serotype disease has remained stable, in both children and older adults (*10*). Clarification of the reasons for differences seen in studies from the United States and United Kingdom is needed because serotype replacement is a threat to the effectiveness of current vaccine programs, and PCV scheduling remains a policy decision area. It remains unclear why such differences occur; however, several factors might be involved, such as methods differences in surveillance approaches, exposure to pneumococcal transmission, risk factor profiles between patient populations, and serotype interactions (*11*). Small differences in carriage prevalence and clonal lineages might result in greatly different rates of IPD because serotype-specific invasiveness varies by orders of magnitude.

In England, the UK Health Security Agency conducts national IPD surveillance, which includes limited data on noninvasive pneumococcal disease, clinical phenotype, and disease severity (*5*–*7*). Evidence from a large pneumococcal pneumonia cohort in Nottingham, UK, suggests there are relatively few differences between patients with PCV13 and non-PCV13 serotype respiratory infections (*12*). Nevertheless, changing serotype distribution could result in changes in pneumococcal disease phenotypes, which might have implications for use of available polyvalent serotype-specific pneumococcal vaccines. Furthermore, the COVID-19 pandemic has disrupted the epidemiology of multiple respiratory infections (*13*,*14*) and provided new insights into virus–bacteria–host interactions. Many countries, including the United Kingdom, implemented measures such as social distancing and school closures that were intended to decrease SARS-CoV-2 transmission and alleviate pressure on healthcare services (*15*). Those measures reduced the transmission of other respiratory pathogens (*14*), but it is unclear to what extent they disrupted pneumococcal transmission (*16*,*17*). The measures might also have caused changes in serotype distribution of pneumococcal infection and disease.

In this retrospective cohort study conducted at 3 large National Health Service (NHS) hospitals, which represent all secondary care provision within a defined geographic area, we examined trends in pneumococcal serotype distribution in adults after PCV7 and PCV13 implementation into the childhood immunization program and the effect of the COVID-19 pandemic over the first 3 years. We report confirmed pneumococcal disease incidence during 2006–2022, both overall and by vaccine serotypes, and assess trends in severity of pneumococcal disease in hospitalized adults.

## Materials and Methods

### Study Design

This study was approved by the UK Health Research Authority (IRAS 265437). We conducted a retrospective cohort study including all patients >16 years of age who were admitted to any of three large UK NHS hospitals in southwest England: University Hospitals Bristol and Weston, North Bristol, and The Royal United Hospital (Bath) NHS Trusts. The study covered the period January 1, 2006‒December 31, 2022, and included patients who had a confirmed microbiologic diagnosis of pneumococcal infection. Those hospitals provide all secondary care within a defined geographic area with 100,000 unplanned adult admissions annually, including the regional cardiothoracic, pleural, respiratory specialist, and general medical and respiratory services.

We identified eligible cases retrospectively by searching the Laboratory Information Management System database (Clinisys WinPath Enterprise). We confirmed identification of *S. pneumoniae* by using culture or PCR from a sterile site at a central laboratory by using standard microbiologic techniques. Culture was confirmed by using API-20 Strep (bioMérieux) or matrix-assisted laser desorption/ionization time-of-flight) mass spectrometry (Bruker). A positive result on pneumococcal urinary antigen test (UAT) (Binax) was also considered confirmation of pneumococcal infection. Patients were included if they tested positive on any or all tests.

We linked confirmed cases with the UK Health Security Agency (UKHSA) national reference laboratory database to obtain serotype data, which we collected at the end of the study to avoid risk for bias in clinical data collection. We reviewed clinical records at each hospital and recorded data in a standardized manner, including laboratory and radiologic investigations. We recorded patient observations within 24 hours of when care was sought for a pneumococcal infection, and established vaccination status by using electronically linked general practitioner records. We calculated the CURB65 severity score (confusion, blood urea nitrogen, respiratory rate, blood pressure, and age >65 years) (https://www.mdcalc.com/calc/324/curb-65-score-pneumonia-severity) at admission for each clinical episode and recorded clinical outcomes, including length of hospitalization and intensive care unit (ICU) admission. We determined inpatient death (i.e., patient death before discharge) through review of the medical records and used it as a marker of case-fatality. All adult patients were managed at the discretion of the admitting clinical team.

### Case Definitions

Total pneumococcal disease included all positive cases, whether identified by sterile site culture/PCR or positive UAT result with clinical confirmation of sterile site infection (i.e., IPD) or noninvasive pneumococcal disease (positive UAT result only). Total cases where the serotype was identified are referred to as serotype-known disease: pneumococcal serotypes were further grouped by vaccine serotypes: PCV7, PCV13–7 (PCV 13 minus PCV7 serotypes, PCV15–13, PCV20–15, PCV20–13, and serotypes not contained in a PCV (non-PCV) (Appendix). The primary infection site was derived from the managing clinician’s diagnosis. Respiratory infection included both consolidative infection (i.e., pneumonia) according to British Thoracic Society/National Institute for Health and Care Excellence (BTS/NICE) guidelines (*18*) and nonpneumonic lower respiratory tract infection.

### Statistical Analysis

Data are reported as medians and interquartile ranges for continuous variables or means and SDs where the distributions were confirmed to be normal by using the Anderson Darling normality test. Categorical variables are presented as counts and percentages. We calculated multinomial CIs by using the Wilson score interval method for binomial percentages (*19*), which we tested on simulated data and found to be well calibrated for this problem. We compared baseline characteristics by using the Fisher exact test for categorical variables, the 2-sample Kolmogorov-Smirnov test for nonparametric continuous variables, the Wilcoxon rank-sum test for score variables, or the 2-sided Student t-test for parametric continuous variables. There were minimal missing data, and only for categorical variables. When these data were present, they were included as a separate category before statistical testing.

We performed multinomial time series analysis by fitting a single-hidden-layer neural network and using the nnet package (*20*) in R (The R Foundation for Statistical Research) with a time-varying natural spline term for class probabilities and a knot point for each class every 2 years. We performed binomial time series analysis by using a maximum-likelihood approach with local polynomial regression and a logistic link function assuming that the count of positive and negative results are a quasi-binomially distributed quantity in the R package locfit according to the methods of Loader et al. (*21*) with a bandwidth equivalent to 2 years’ worth of data and a polynomial of degree 2.

We obtained yearly estimates for the >16 years of age population of clinical commissioning groups of NHS Bath and North East Somerset and NHS Bristol, North Somerset, and South Gloucestershire regions from NHS Digital. We interpolated yearly point estimates to daily estimates by using a local polynomial regression and estimated pneumococcal disease incidence as the rate of monthly admission counts by using a local polynomial regression assuming a quasi-Poisson distribution, logarithmic link function, and the methods of Loader et al. (*21*). We expressed admission rates as disease cases per 1,000 person-years by using interpolated population estimates. We performed all analyses by using R version 4.2.

## Results

During 2006–2022, we identified 3,719 adults (median age 66.1 years, interquartile range 50.2–78.9 years) who had pneumococcal disease. Of those persons, 1,840 (49.5%) were male, 1,879 (50.5%) were female, 1,621 (43.6%) had positive blood cultures, 2,379 (64.0%) were UAT positive, and 15 (0.4%) were PCR positive. Among the cases, 1,686 (45.3%) were IPD and 2,033 (54.7%) were non-IPD. Respiratory infection accounted for 92.3% (3,436/3,719) of all cases of pneumococcal disease, 84.2% of IPD, and 99.2% of noninvasive pneumococcal disease (Table 1). Pneumococcal serotype was available for 1,501 (40%) cases. The demographics and clinical characteristics of patients with known-serotype pneumococcal disease were similar to those with unknown serotype infection (Appendix).

**Table 1:** Characteristics of patients hospitalized with confirmed pneumococcal infection, Bristol, UK, 2006–2022*

Characteristic	Invasive disease, n = 1,686	Noninvasive disease, n = 2,033	p value
Age, y, median (IQR)	65.9 (50.8‒79)	66.3 (50‒78.8)	0.74
Sex			
M	847 (50.2)	993 (48.8)	0.41
F	839 (49.8)	1,040 (51.2)	
Serotype status			
Serotype identified	1,501 (89.0)	0	<0.001
No serotype	185 (11.0)	2,033 (100.0)	
Smoker			
Nonsmoker	495 (29.4)	597 (29.4)	0.95
Ex-smoker	680 (40.3)	829 (40.8)	
Current smoker	511 (30.3)	607 (29.9)	
Charlson Comorbidity Index, median (IQR)	4 (1‒6)	4 (1‒6)	0.31
Test type			
Blood culture only	1,325 (78.6)	0 (0)	<0.001
UAT only	51 (3.0)	2,033 (100.0)	
Blood culture and UAT	295 (17.5)	0	
CSF PCR	8 (0.5)	0	
Blood PCR	7 (0.4)	0	
Infection site			
Lung	1,419 (84.2)	2,017 (99.2)	<0.001
Meningitis	172 (10.2)	0	
Septic arthritis	36 (2.1)	0	
ENT	15 (0.9)	2 (0.1)	
Other	44 (2.6)	14 (0.7)	
PPV23 vaccination, time before illness			
None	967 (57.4)	1,171 (57.6)	0.99
<6 mo	60 (3.6)	72 (3.5)	
>6 mo	659 (39.1)	789 (38.8)	
Missing	0	1 (0)	
Noninvasive ventilation	39 (2.3)	103 (5.1)	<0.001
Intubation	236 (14.0)	234 (11.5)	0.026
Inpatient death†	255 (15.1)	253 (12.4)	0.019

*Values are no. (%) except as indicated. Significance was determined by using the Fisher exact test for categorical variables, the 2-sample Kolmogorov-Smirnov test (continuous variables), and the 2-sample Wilcoxon rank-sum test (continuous variables). Normality of distributions determined by using the Anderson-Darling normality test. CSF, cerebrospinal fluid; ENT, ear, nose, and throat; IQR, interquartile range; PPV23, 23-valent pneumococcal polysaccharide vaccine; UAT, urine antigen test. †Determined though review of medical records (i.e., death before discharge from hospital).

**Figure 1:**
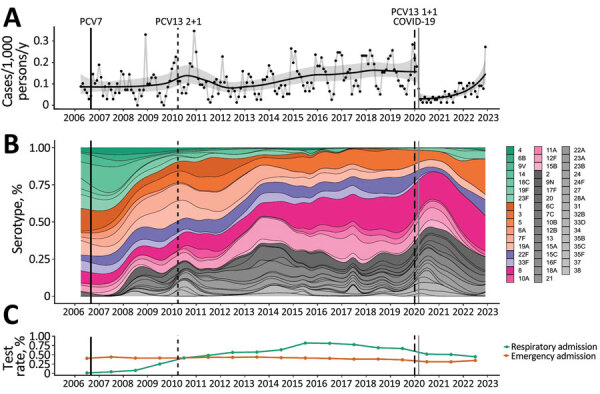
Distribution of pneumococcal serotypes in hospitalized patients, Bristol and Bath, UK, 2006–2022. A) Incidence of blood culture‒positive pneumococcal disease. Data shown include only invasive disease; for comparison with noninvasive disease, see the Appendix. Black dots indicate monthly observations, black line indicates a binomial time series model, and gray shading indicate 95% CIs. Population estimates are provided in the Appendix. B) Multinomial percentage of pneumococcal serotypes in the invasive pneumococcal disease population with known serotype. Green bars indicate 7-valent PCV (PCV7) serotypes; orange, PCV13–7 (PCV13 minus PCV7 serotypes); purple, PCV15–13; pink, PCV20–15; gray, serotypes not contained in PCV vaccines. Individual serotypes are shown in the legend on the panel. C) Testing rates for blood cultures with respect to acute admissions and BinaxNOW (https://www.abbott.com) tests with respect to acute respiratory admissions. Estimates are presented at the midyear time point, relative to the data they represent. For all panels, vertical lines indicate introduction of PCV7 (solid black), PCV13 (2 + 1 schedule) (thin black dashed line), PCV13 (1 + 1 schedule) (thick black dashed line) into the UK childhood vaccination program and the beginning of large hospital admissions in Bristol caused by SARS-CoV-2 (solid gray). PCV, pneumococcal conjugate vaccine.

The incidence of IPD among hospitalized adults showed a slight upward trend during 2006‒late 2019 (Figure 1, panel A). During the early part of the COVID-19 pandemic in 2020, pneumococcal disease incidence suddenly decreased. However, during 2022, incidence gradually increased so that by December 2022 it had returned to a level similar to that observed before the COVID-19 pandemic (Figure 1, panel A). Noninvasive pneumococcal disease showed a broadly similar pattern but is harder to interpret because of changing BinaxNOW testing patterns over the study period (Figure 1, panel C; Appendix Figures 3, 4). PCV7 serotype disease decreased from 29.4% (95% CI 24.1%–35.4%) in 2006–2009 to 7.0% (95% CI 3.7%–12.7%) of serotype-known disease in 2021–2022, and PCV7-serotypes caused minimal disease from mid-2017 until their re-emergence during the COVID-19 pandemic. PCV13–7 disease represented 34.3% (95% CI 28.6%–40.4%) of serotype-known disease in 2006–2009, decreased slightly to 29.3% (95% CI 25.6%–33.3%) by 2010–2015, and continued to decrease to 21.7% (95% CI 15.5%–29.6%) in 2021–2022, with serotypes 1, 3, and 19A persisting. In contrast, the percentage of PCV20–13 and non-PCV serotype disease increased during this period (Figure 1, panel B).

**Figure 2:**
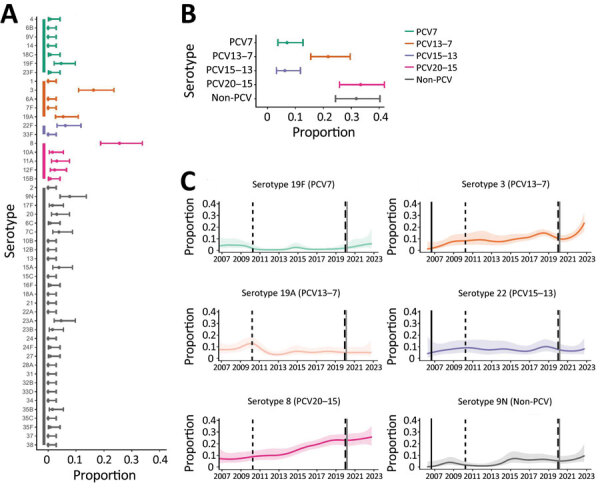
Distribution of pneumococcal serotypes in hospitalized patients after emergence of SARS-CoV-2. A, B) Proportion of pneumococcal disease attributable to each serotype (A) and PCV vaccine group (B) from 129 case-patients in Bristol, UK, hospitalized during January 1, 2021‒December 31, 2022. C) Binomial (one versus others) time series models showing the percentage of disease during January 2006‒December 2022 attributable to each of the 6 most common pneumococcal serotypes. Solid lines indicate smoothed point estimates; error bars/shaded areas indicate 95% CIs. The 95% CIs in panels A and B do not coincide exactly with those in panel C because they represent different length time periods and use different models. Additional data and time points are available in Table 2. PCV, pneumococcal conjugate vaccine.

Although blood culture testing rates remained stable over time, there were changes in UAT testing rates. The decreased pneumococcal disease incidence observed during the COVID-19 pandemic occurred without an equivalent decrease in BinaxNOW and blood culture testing over the same time period (Figure 1, panel C). After SARS-CoV-2 emergence, the most commonly identified serotypes were 3, 8, 9N, 19F, 19A and 22F (Figure 2, panels A, B). In 2022, 6.2% (95% CI 3.2%–11.8%) of disease was attributable to PCV15–13, 33.3% (95% CI 25.8%–41.8%) to PCV20–15, and 31.8% (95% CI 24.4%–40.2%) to non-PCV serotypes; a major percentage of disease arose from serotypes in current PCVs (Figure 2). Among known-serotype cases, PCV7 serotypes represented 7.0% (95% CI 3.7%–12.7%) and PCV13–7 serotypes 21.7% (95% CI 15.5%–29.6%) of cases (Table 2). Overall, the percentage of disease caused by 19F, 19A, and 22F remained relatively stable throughout the study, and an expansion of serotypes 3 and 8 disease occurred, along with a slight increase in serotype 9N (Figure 2, panel C).

**Table 2:** Case number and percentage of disease attributable to each pneumococcal serotype, by vaccine group, Bristol. UK, January 2021‒December 2022*

Vaccine and serotype	2006–2009			2010–2015			2016–2019			2020			2021–2022	
	Cases	% (95% CI)		Cases	% (95% CI)		Cases	% (95% CI)		Cases	% (95% CI)		Cases	% (95% CI)
PCV7														
Subtotal	73/248	29.4 (24.1‒35.4)		38/536	7.1 (5.2‒9.6)		8/534	1.5 (0.8‒2.9)		1/54	1.9 (0.3‒9.8)		9/129	7.0 (3.7‒12.7)
4	8/248	3.2 (1.6‒6.2)		5/536	0.9 (0.4‒2.2)		3/534	0.6 (0.2‒1.6)		0/54	0.0 (0.0‒6.6)		1/129	0.8 (0.1‒4.3)
6B	6/248	2.4 (1.1‒5.2)		4/536	0.7 (0.3‒1.9)		0/534	0.0 (0.0‒0.7)		0/54	0.0 (0.0‒6.6)		0/129	0.0 (0.0‒2.9)
9V	8/248	3.2 (1.6‒6.2)		2/536	0.4 (0.1‒1.4)		0/534	0.0 (0.0‒0.7)		0/54	0.0 (0.0‒6.6)		0/129	0.0 (0.0‒2.9)
14	25/248	10.1 (6.9‒14.5		6/536	1.1 (0.5‒2.4)		2/534	0.4 (0.1‒1.4)		0/54	0.0 (0.0‒6.6)		0/129	0.0 (0.0‒2.9)
18C	5/248	2.0 (0.9‒4.6)		3/536	0.6 (0.2‒1.6)		0/534	0.0 (0.0‒0.7)		0/54	0.0 (0.0‒6.6)		1/129	0.8 (0.1‒4.3)
19F	8/248	3.2 (1.6‒6.2)		5/536	0.9 (0.4‒2.2)		3/534	0.6 (0.2‒1.6)		1/54	1.9 (0.3‒9.8)		6/129	4.7 (2.1‒9.8)
23F	13/248	5.2 (3.1‒8.8)		13/536	2.4 (1.4‒4.1)		0/534	0.0 (0.0‒0.7)		0/54	0.0 (0.0‒6.6)		1/129	0.8 (0.1‒4.3)
PCV13–7														
Subtotal	85/248	34.3 (28.6‒40.4)		157/536	29.3 (25.6‒33.3)		105/534	19.7 (16.5‒23.2)		5/54	9.3 (4.0‒19.9)		28/129	21.7 (15.5‒29.6)
1	22/248	8.9 (5.9‒13.1)		30/536	5.6 (3.9‒7.9)		3/534	0.6 (0.2‒1.6)		0/54	0.0 (0.0‒6.6)		0/129	0.0 (0.0‒2.9)
3	12/248	4.8 (2.8‒8.3)		43/536	8.0 (6.0‒10.6)		63/534	11.8 (9.3‒14.8)		4/54	7.4 (2.9‒17.6)		21/129	16.3 (10.9‒23.6)
6A	9/248	3.6 (1.9‒6.8)		1/536	0.2 (0.0‒1.0)		0/534	0.0 (0.0‒0.7)		0/54	0.0 (0.0‒6.6)		0/129	0.0 (0.0‒2.9)
7F	22/248	8.9 (5.9‒13.1)		49/536	9.1 (7.0‒11.9)		10/534	1.9 (1.0‒3.4)		0/54	0.0 (0.0‒6.6)		0/129	0.0 (0.0‒2.9)
19A	20/248	8.1 (5.3‒12.1)		34/536	6.3 (4.6‒8.7)		29/534	5.4 (3.8‒7.7)		1/54	1.9 (0.3‒9.8)		7/129	5.4 (2.7‒10.8)
PCV13	158/248	63.7 (57.6‒69.4)		195/536	36.4 (32.4‒40.5)		113/534	21.2 (17.9‒24.8)		6/54	11.1 (5.2‒22.2)		37/129	28.7 (21.6‒37.0)
Non-PCV13	90/248	36.3 (30.6‒42.4)		341/536	63.6 (59.5‒67.6)		421/534	78.8 (75.2‒82.1)		48/54	88.9 (77.8‒94.8)		92/129	71.3 (63.0‒78.4)
PCV15–13														
Subtotal	29/248	11.7 (8.3‒16.3)		63/536	11.8 (9.3‒14.8)		62/534	11.6 (9.2‒14.6)		4/54	7.4 (2.9‒17.6)		8/129	6.2 (3.2‒11.8)
22F	17/248	6.9 (4.3‒10.7)		44/536	8.2 (6.2‒10.8)		42/534	7.9 (5.9‒10.5)		4/54	7.4 (2.9‒17.6)		8/129	6.2 (3.2‒11.8)
33F	12/248	4.8 (2.8‒8.3)		19/536	3.5 (2.3‒5.5)		20/534	3.7 (2.4‒5.7)		0/54	0.0 (0.0‒6.6)		0/129	0.0 (0.0‒2.9)
PCV15	187/248	75.4 (69.7‒80.3)		258/536	48.1 (43.9‒52.4)		175/534	32.8 (28.9‒36.9)		10/54	18.5 (10.4‒30.8)		45/129	34.9 (27.2‒43.4)
Non-PCV15	61/248	24.6 (19.7‒30.3)		278/536	51.9 (47.6‒56.1)		359/534	67.2 (63.1‒71.1)		44/54	81.5 (69.2‒89.6)		84/129	65.1 (56.6‒72.8)
PCV20–15														
Subtotal	38/248	15.3 (11.4‒20.3)		133/536	24.8 (21.3‒28.6)		221/534	41.4 (37.3‒45.6)		17/54	31.5 (20.7‒44.7)		43/129	33.3 (25.8‒41.8)
8	18/248	7.3 (4.6‒11.2)		62/536	11.6 (9.1‒14.6)		113/534	21.2 (17.9‒24.8)		9/54	16.7 (9.0‒28.7)		33/129	25.6 (18.8‒33.7)
10A	0/248	0.0 (0.0‒1.5)		11/536	2.1 (1.1‒3.6)		19/534	3.6 (2.3‒5.5)		2/54	3.7 (1.0‒12.5)		2/129	1.6 (0.4‒5.5)
11A	6/248	2.4 (1.1‒5.2)		7/536	1.3 (0.6‒2.7)		23/534	4.3 (2.9‒6.4)		2/54	3.7 (1.0‒12.5)		4/129	3.1 (1.2‒7.7)
12F	10/248	4.0 (2.2‒7.3)		48/536	9.0 (6.8‒11.7)		57/534	10.7 (8.3‒13.6)		3/54	5.6 (1.9‒15.1)		3/129	2.3 (0.8‒6.6)
15B	4/248	1.6 (0.6‒4.1)		5/536	0.9 (0.4‒2.2)		9/534	1.7 (0.9‒3.2)		1/54	1.9 (0.3‒9.8)		1/129	0.8 (0.1‒4.3)
PCV20–13	67/248	27.0 (21.9‒32.9)		196/536	36.6 (32.6‒40.7)		283/534	53.0 (48.8‒57.2)		21/54	38.9 (27.0‒52.2)		51/129	39.5 ([31.5‒48.2)
PCV20	225/248	90.7 (86.5‒93.7)		391/536	72.9 (69.0‒76.5)		396/534	74.2 (70.3‒77.7)		27/54	50.0 (37.1‒62.9)		88/129	68.2 (59.8‒75.6)
Non-PCV	23/248	9.3 (6.3‒13.5)		145/536	27.1 (23.5‒31.0)		138/534	25.8 (22.3‒29.7)		27/54	50.0 (37.1‒62.9)		41/129	31.8 (24.4‒40.2)
2	0/248	0.0 (0.0‒1.5)		0/536	0.0 (0.0‒0.7)		1/534	0.2 (0.0‒1.1)		0/54	0.0 (0.0‒6.6)		0/129	0.0 (0.0‒2.9)
9N	5/248	2.0 (0.9‒4.6)		20/536	3.7 (2.4‒5.7)		27/534	5.1 (3.5‒7.3)		3/54	5.6 (1.9‒15.1)		10/129	7.8 (4.3‒13.7)
17F	0/248	0.0 (0.0‒1.5)		0/536	0.0 (0.0‒0.7)		6/534	1.1 (0.5‒2.4)		0/54	0.0 (0.0‒6.6)		2/129	1.6 (0.4‒5.5)
20	1/248	0.4 (0.1‒2.2)		4/536	0.7 (0.3‒1.9)		9/534	1.7 (0.9‒3.2)		4/54	7.4 (2.9‒17.6)		4/129	3.1 (1.2‒7.7)
6C	2/248	0.8 (0.2‒2.9)		23/536	4.3 (2.9‒6.4)		3/534	0.6 (0.2‒1.6)		1/54	1.9 (0.3‒9.8)		1/129	0.8 (0.1‒4.3)
7C	0/248	0.0 (0.0‒1.5)		0/536	0.0 (0.0‒0.7)		5/534	0.9 (0.4‒2.2)		1/54	1.9 (0.3‒9.8)		5/129	3.9 (1.7‒8.8)
10B	0/248	0.0 (0.0‒1.5)		0/536	0.0 (0.0‒0.7)		2/534	0.4 (0.1‒1.4)		0/54	0.0 (0.0‒6.6)		0/129	0.0 (0.0‒2.9)
12B	0/248	0.0 (0.0‒1.5)		3/536	0.6 (0.2‒1.6)		0/534	0.0 (0.0‒0.7)		0/54	0.0 (0.0‒6.6)		0/129	0.0 (0.0‒2.9)
13	0/248	0.0 (0.0‒1.5)		1/536	0.2 (0.0‒1.0)		1/534	0.2 (0.0‒1.1)		0/54	0.0 (0.0‒6.6)		0/129	0.0 (0.0‒2.9)
15A	2/248	0.8 (0.2‒2.9)		24/536	4.5 (3.0‒6.6)		20/534	3.7 (2.4‒5.7)		2/54	3.7 (1.0‒12.5)		5/129	3.9 (1.7‒8.8)
15C	3/248	1.2 (0.4‒3.5)		2/536	0.4 (0.1‒1.4)		1/534	0.2 (0.0‒1.1)		0/54	0.0 (0.0‒6.6)		0/129	0.0 (0.0‒2.9)
16F	2/248	0.8 (0.2‒2.9)		8/536	1.5 (0.8‒2.9)		11/534	2.1 (1.2‒3.7)		4/54	7.4 (2.9‒17.6)		1/129	0.8 (0.1‒4.3)
18A	0/248	0.0 (0.0‒1.5)		0/536	0.0 (0.0‒0.7)		1/534	0.2 (0.0‒1.1)		0/54	0.0 (0.0‒6.6)		0/129	0.0 (0.0‒2.9)
21	0/248	0.0 (0.0‒1.5)		1/536	0.2 (0.0‒1.0)		1/534	0.2 (0.0‒1.1)		0/54	0.0 (0.0‒6.6)		0/129	0.0 (0.0‒2.9)
22A	1/248	0.4 (0.1‒2.2)		0/536	0.0 (0.0‒0.7)		1/534	0.2 (0.0‒1.1)		0/54	0.0 (0.0‒6.6)		0/129	0.0 (0.0‒2.9)
23A	1/248	0.4 (0.1‒2.2)		14/536	2.6 (1.6‒4.3)		8/534	1.5 (0.8‒2.9)		2/54	3.7 (1.0‒12.5)		6/129	4.7 (2.1‒9.8)
23B	0/248	0.0 (0.0‒1.5)		11/536	2.1 (1.1‒3.6)		6/534	1.1 (0.5‒2.4)		3/54	5.6 (1.9‒15.1)		2/129	1.6 (0.4‒5.5)
24	0/248	0.0 (0.0‒1.5)		0/536	0.0 (0.0‒0.7)		1/534	0.2 (0.0‒1.1)		0/54	0.0 (0.0‒6.6)		0/129	0.0 (0.0‒2.9)
24F	0/248	0.0 (0.0‒1.5)		6/536	1.1 (0.5‒2.4)		9/534	1.7 (0.9‒3.2)		1/54	1.9 (0.3‒9.8)		1/129	0.8 (0.1‒4.3)
27	0/248	0.0 (0.0‒1.5)		0/536	0.0 (0.0‒0.7)		2/534	0.4 (0.1‒1.4)		0/54	0.0 (0.0—6.6)		1/129	0.8 (0.1‒4.3)
28A	0/248	0.0 (0.0‒1.5)		0/536	0.0 (0.0‒0.7)		0/534	0.0 (0.0‒0.7)		1/54	1.9 (0.3—9.8)		0/129	0.0 (0.0‒2.9)
31	3/248	1.2 (0.4‒3.5)		1/536	0.2 (0.0‒1.0)		2/534	0.4 (0.1‒1.4)		1/54	1.9 (0.3—9.8)		0/129	0.0 (0.0‒2.9)
32B	0/248	0.0 (0.0‒1.5)		1/536	0.2 (0.0‒1.0)		1/534	0.2 (0.0‒1.1)		0/54	0.0 (0.0—6.6)		0/129	0.0 (0.0‒2.9)
33D	0/248	0.0 (0.0‒1.5)		0/536	0.0 (0.0‒0.7)		2/534	0.4 (0.1‒1.4)		0/54	0.0 (0.0—6.6)		0/129	0.0 (0.0‒2.9)
34	0/248	0.0 (0.0‒1.5)		0/536	0.0 (0.0‒0.7)		2/534	0.4 (0.1‒1.4)		0/54	0.0 (0.0—6.6)		0/129	0.0 (0.0‒2.9)
35B	1/248	0.4 (0.1‒2.2)		8/536	1.5 (0.8‒2.9)		11/534	2.1 (1.2‒3.7)		1/54	1.9 (0.3—9.8)		2/129	1.6 (0.4‒5.5)
35C	0/248	0.0 (0.0‒1.5)		2/536	0.4 (0.1‒1.4)		0/534	0.0 (0.0‒0.7)		0/54	0.0 (0.0‒6.6)		0/129	0.0 (0.0‒2.9)
35F	0/248	0.0 (0.0‒1.5)		10/536	1.9 (1.0‒3.4)		3/534	0.6 (0.2‒1.6)		2/54	3.7 (1.0‒12.5)		1/129	0.8 (0.1‒4.3)
37	0/248	0.0 (0.0‒1.5)		1/536	0.2 (0.0‒1.0)		0/534	0.0 (0.0‒0.7)		1/54	1.9 (0.3‒9.8)		0/129	0.0 (0.0‒2.9)
38	2/248	0.8 (0.2‒2.9)		5/536	0.9 (0.4‒2.2)		2/534	0.4 (0.1‒1.4)		0/54	0.0 (0.0‒6.6)		0/129	0.0 (0.0‒2.9)

*Values are no. positive/no. tested except as indicated. PCV, pneumococcal conjugate vaccination.

**Figure 3:**
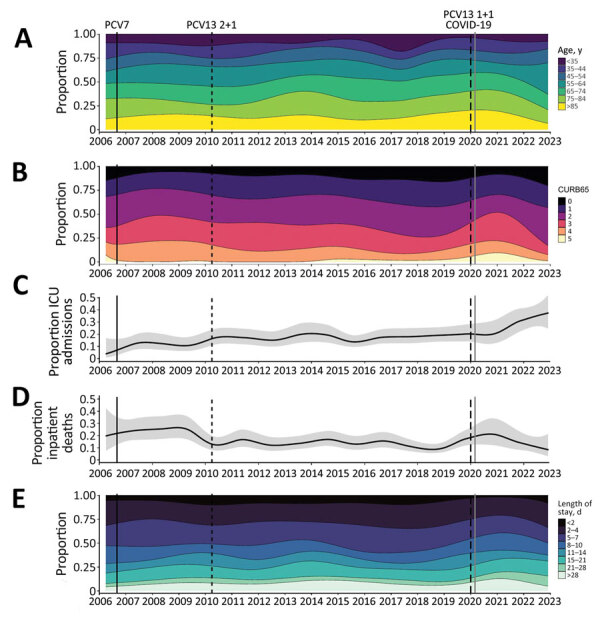
Severity of manifestations and outcome of invasive pneumococcal disease in hospitalized adults over time, Bristol, UK. A) Age categories; B) CURB65 scores; C) ICU admissions; D) inpatient deaths; E) length of stay in a hospital. For panels A, B, and E, categories are shown in the legend adjacent to each panel. The solid line in the multinomial time series models shown in panels C and D indicates binomial time series models; gray areas indicate 95% CIs. Across all panels, vertical lines indicate dates of 7-valent PCV (PCV7) (solid black) and PCV13 2 + 1 (thin black dashed line) and 1 + 1 (thick dash blacked line) schedule vaccine introduction and SARS-CoV-2 emergence (solid gray). Results for noninvasive pneumococcal disease are shown in the Appendix. ICU, intensive care unit; PCV, pneumococcal conjugate vaccine.

Before the emergence of SARS-CoV-2, the age of patients admitted with IPD increased over time (Figure 3, panel A) and disease severity decreased, as shown by the average CURB65 severity scores at admission (Figure 3, panel B). ICU admission rates increased in earlier years and then plateaued, inpatient deaths decreased, and length of hospital admission remained broadly stable (Figure 3, panels C‒E). Those trends were disrupted during the early stages of the SARS-CoV-2 pandemic and largely reverted to their previous trajectories in 2021–2022. The percentage of patients 45–55 years of age trended upwards compared with the prepandemic period, and the percentage of IPD case-patients requiring ICU admission also increased, but those trends were based on small numbers. We could not account for the increase in ICU admissions seen after SARS-CoV-2 emergence through change in bed availability or clinical care pathways.

## Discussion

This study examining hospitalized adults with pneumococcal disease in Bristol and Bath, UK, encompasses 17 years of PCV implementation into the UK childhood vaccination program and includes 3 years of the COVID-19 pandemic. Although the percentage of IPD caused by PCV serotypes has decreased across all age groups, a major fraction of adult pneumococcal disease is still caused by PCV7 (7.0% [95% CI 3.7%–12.7%]) and PCV13–7 (21.7% [95% CI 15.5%–29.6%]) serotypes (*5**,**22*). In addition, the percentage of adult disease caused by those serotypes has increased since COVID-19 pandemic restrictions began in March 2020, which also coincided with a change in the UK childhood immunization schedule from 3 doses to 2 doses of PCV13 since April 2020. The persistence of PCV13 serotype and PCV7 serotype disease in adults in this study occurred in the context of a longstanding, high-coverage national childhood vaccination program. This persistence suggests that, although giving PCVs to most children has had some indirect effects, those effects do not completely protect adults from PCV serotype disease.

The persistence of some PCV13 serotypes is concerning because they are associated with more severe disease and deaths in adults. In addition, we have observed large increases in the percentage of non-PCV13 serotype disease since PCV13 implementation, consistent with national surveillance throughout the UK and Europe (*5**‒**7*,*12*,*22*). During 2016–2019, non-PCV13 serotypes caused 78.8% (95% CI 75.2%–82.1%) of cases among persons who had identified serotypes; during 2021–2022 that percentage was 71.3% (95% CI 63.0%–78.4%). During 2021–2022, PCV20–13 serotypes accounted for 39.5% (95% CI 31.5%–48.2%) of adult disease with identified serotypes.

Despite indirect effects operating to some degree, PCV serotypes continue to cause adult disease that is severe enough to require hospital admission. Although PCV7 serotypes remain rare causes of adult pneumococcal disease, serotype 19F has reemerged. We found that most residual PCV serotype disease is caused by PCV13–7 serotypes, especially serotypes 3 and 19A, observations that are consistent with other reports (*5*,*7*,*12*,*22*). The continued circulation of serotype 3, especially after the pandemic restrictions were lifted, is critical because this serotype is associated with more severe disease, especially in older adults. Ongoing circulation might, in part, be caused by clade II expansion after a clade distribution shift (*23*). Serotype 19A is also of concern because it has previously been associated with high antimicrobial drug resistance rates in some countries (*24*). 

Indirect effects of PCVs are believed to occur by reduction in nasopharyngeal carriage and density of vaccine serotypes in vaccinated children, which interrupts transmission to both vaccinated and unvaccinated contacts (*2*–*4*). Generally, sustained high PCV coverage in children is assumed to result in reliable indirect effects in adults: the UK 1 + 1 PCV13 childhood immunization schedule relies, to a degree, on those indirect effects to protect infants who only receive 1 priming PCV dose before their 1-year booster (*25*). However, we cannot explain the recent percentage increase in PCV13 serotype disease we observed by decreasing pediatric PCV vaccination rates. In the United Kingdom, vaccination is offered free of charge and is associated with high uptake; PCV uptake was >90% for the first dose by 12 months of age and >94% for the second dose by 24 months of age in southwestern England in 2021–2022 (*26*).

A concerning finding is the high percentage of cases that are not vaccine preventable: during 2016–19, PCV20–13 and non-PCV serotypes represented 53.0% (95% CI 48.8%–57.2%) and 25.8% (95% CI 22.3%–29.7%) of cases, respectively; in 2021‒2022, those percentages of 39.5% (95% CI 31.5%–48.2%) and 31.8% (95% CI 24.4%–40.2%). In 2021–22, PCV15 serotypes represented 34.9% (95% CI 27.2%–43.4%) of IPD cases, PCV20 serotypes 68.2% (95% CI 59.8%–75.6%) and non-PCV serotypes 31.8% (95% CI 24.4%–40.2%). Although serotype replacement has been observed in Europe and serotype distribution change in the United States (*5*–*7*,*21*,*27*,*28*), reports from the United States describe consistently stable rates of non-PCV13 serotype IPD after childhood PCV13 introduction (*29*). Serotype replacement might occur through expansion of non-PCV strains or capsular switching of previously vaccine-preventable strains, in which recombination at the capsular gene locus results in serotype change (*30*). In concordance with other studies, we found serotype 8 disease increasing, along with 23A and 9N (*5*,*7*,*12*).

We observed a disproportionate decrease in pneumococcal disease incidence early in the COVID-19 pandemic, without a concomitant decrease in BinaxNOW and blood culture testing rates. This finding might have been caused by reduced circulation and transmission of many respiratory pathogens, including pneumococcus, because of social distancing policies (*13*,*14*,*16*) breaking transmission chains and affecting host‒pathogen‒pathogen interactions, so that severe pneumococcal infection became less common. Preceding viral infection is a major risk factor for pneumonia, and the relationships between influenza and respiratory syncytial virus and pneumococcus are well described (*31*,*32*). Pneumococcus might also modulate host immune responses to SARS-CoV-2 (*33*). Prospective surveillance found that Europe experienced major and sustained reductions in invasive disease caused by pneumococcus, *Haemophilus influenzae*, and *Neisseria meningitidis* in early 2020 (*34*). Our study confirms that reduced testing practices in hospitals do not explain such reductions.

However, changes in healthcare use might explain the incidence decreases that occurred. For example, susceptible patients had fewer admissions to secondary care during the pandemic (*35*). An increased admission threshold or avoidance of secondary care would increase community-level management of cases and reduce case-patients who have pneumococcal disease seeking treatment and consequently being diagnosed in hospitals. Because fewer investigations are performed in community-level care, such cases would be missed by surveillance that is reliant on microbiologic testing.

By combining microbiologic results with clinical data, we are able to report on both disease epidemiology and severity over time. We found some evidence of more severe disease, especially after SARS-CoV-2 emergence. The observed disruption to the broadly stable previous trends in disease severity during the early part of the SARS-CoV-2 pandemic might be a result of observation bias, a consequence of the pandemic, or both. Although ICU admission rates increased for pneumococcal disease, admission CURB65 severity scores were lower, consistent with the 2018 BTS national pneumonia audit, which also reported decreased admission CURB65 scores (*36*). This apparent discrepancy might be explained by changes in patients or their treatment (e.g., older patients with more severe disease might be less likely to receive ICU care than younger patients because of clinical prognostic decision making). Other recent changes in treatment, such as increased availability of positive pressure support or earlier antimicrobial drug administration, might also have changed patient courses and outcomes, including severity and death. Major changes in pneumococcal infection severity have occurred since 2020, including an increased percentage of adults 45–55 years of age who required hospital treatment. The recent increase in cases is probably related to relaxation of COVID-19 restrictions. Recent studies highlight the critical role of respiratory viruses (e.g., respiratory syncytial virus, human metapneumovirus, influenza virus) in contributing to pneumococcal disease (*17*). Large, out-of-season viral outbreaks that occurred after easing of COVID-19 restrictions (*37*) are therefore likely to have contributed to the increasing pneumococcal disease rates we report in this study, which will need careful surveillance in future years. Independent of changes in clinical practice, serotype distribution changes might affect patient outcomes and healthcare use as invasive potential varies by serotype.

By combining microbiologic results with clinical data, we are able to report on both disease epidemiology and severity over time. We found some evidence of more severe disease especially after SARS-CoV-2 emergence. The observed disruption to the broadly stable previous trends in disease severity during the early part of the SARS-CoV-2 pandemic might be a result of observation bias, a consequence of the pandemic, or both. Although ICU admission rates increased for pneumococcal disease, we observed a reduction in admission CURB65 severity scores, consistent with the 2018 BTS national pneumonia audit, which also reported decreased admission CURB65 scores (*36*). This apparent discrepancy might be explained by changes in patients or their treatment (e.g., older patients with more severe disease might be less likely to receive ICU care than younger patients because of clinical prognostic decision making). Other recent changes in treatment, such as increased availability of positive pressure support or earlier antimicrobial drug administration, might also have changed patient courses and outcomes, including severity and death. Major changes in pneumococcal infection severity have occurred since 2020, including an increased percentage of adults 45–55 years of age who required hospital treatment. The recent increase in cases is probably related to relaxation of COVID-19 restrictions. Recent studies highlight the critical role of respiratory viruses (e.g., respiratory syncytial virus, human metapneumovirus, influenza virus) in contributing to pneumococcal disease (*17*). Large, out-of-season viral outbreaks that occurred after easing of COVID-19 restrictions (*37*) are therefore likely to have contributed to the increasing pneumococcal disease we report in this study, which will need careful surveillance in future years. Independent of changes in clinical practice, serotype distribution changes might affect patient outcomes and healthcare use as invasive potential varies by serotype.

One strength of this study was that we identified patients who had with pneumococcal infections in a population of ≈1 million adults and report disease over a 17-year period with epidemiologic data supported by detailed clinical information at an individualized patient level. Data on admission numbers and testing practices enabled us to interpret incidence considering these factors, which might otherwise bias the estimates obtained. In addition, linkage with the UKHSA national reference laboratory enabled us to report serotype data whenever available. Our study also reports pneumococcal disease confirmed by UAT; this result was not detected by national surveillance and enabled us to estimate pneumococcal disease incidence and burden more accurately.

One limitation of this study was that UAT does not identify serotype; we were thus unable to report serotype data in 60% of our cohort. However, patients with known and unknown serotype disease had broadly similar demographic and clinical characteristics and outcomes. UAT has a sensitivity of 65%, so test results might remain positive for some time after pneumococcal disease has been treated (*38*). Although the data are concordant with national UKHSA epidemiologic and BTS pneumonia audit data (*36*), providing major reassurance, this regional study might not be representative of other regions or populations. As a retrospective observational study, only information as documented in medical records could be included. Patient treatment decisions might have varied over time and by treating physicians, and systemic changes in patient treatment, healthcare provision, or admission threshold and microbiologic testing practices might have occurred. Those factors might have affected the trends reported in this analysis. Finally, the serotype trends reported in 2020–2022 were based on substantially lower case numbers than those seen before the emergence of SARS-CoV-2. Ongoing monitoring of trends will be useful as disease incidence adjusts after the COVID-19 pandemic.

In conclusion, this study provides evidence that PCV13 serotypes continue to cause pneumococcal disease in adults, suggesting that indirect effects of the childhood PCV program might not lead to additional reductions in the residual burden of those serotypes in adults. After the COVID-19 pandemic, serotype distribution changes have resulted in an increase in adult pneumococcal disease attributable to PCV serotypes, and a major burden of current disease could be directly prevented by introducing PCV20, which has recently been licensed for adults. Ongoing surveillance of adult pneumococcal disease is vital to evaluate vaccine effect and monitor replacement disease. The extent of indirect effects of PCVs should continue to be carefully evaluated and considered in formulating future public health policy recommendations.

Appendix Additional information on serotype distribution and disease severity in adults hospitalized with *Streptococcus pneumoniae* infection, Bristol and Bath, UK, 2006‒2022.
