# Supplementary material for: Serotype Distribution and Disease Severity in Adults Hospitalized with Streptococcus pneumoniae Infection, Bristol and Bath, UK, 2006‒2022
Source: Emerg Infect Dis. 2023 Oct;29(10):1953–64. doi: 10.3201/eid2910.230519 (PMC10521591; doi:10.3201/eid2910.230519)
Supplement: Appendix — Additional information on serotype distribution and disease severity in adults hospitalized with Streptococcus pneumoniae infection, Bristol and Bath, UK, 2006‒2022. [file 23-0519-Techapp-s1.pdf]

Article DOI: https://doi.org/10.3201/eid2910.230519

EID cannot ensure accessibility for Supplemental Materials supplied by authors. Readers who have difficulty accessing supplementary content should contact the authors for assistance.

Serotype Distribution and Disease Severity in Adults Hospitalized with *Streptococcus pneumoniae* Infection, Bristol and Bath, UK, 2006–2022

Appendix

Appendix Table 1. Serotypes contained within each pneumococcal vaccination and vaccine group*.

Vaccination	Serotypes
PPV-23, PneumoVax	1, 2, 3, 4, 5, 6B, 7F, 8, 9N, 9V, 10A, 11A, 12F, 14, 15B, 17F, 18C, 19F, 19A, 20, 22F, 23F, 33F.
PCV-7, Prevenar	4, 6B, 9V, 14, 18C, 19F and 23F
PCV-13, Prevenar13	1, 3, 4, 5, 6A, 6B, 7F, 9V, 14, 18C, 19A, 19F, and 23F
PCV-15, VAXNEUVANCE	1, 3, 4, 5, 6A, 6B, 7F, 9V, 14, 18C, 19A, 19F, 22F, 23F and 33F
PCV-20, Prevenar20	1, 3, 4, 5, 6A, 6B, 7F, 8, 9V,10A, 11A, 12F, 14, 15B, 18C, 19A, 19F, 22F, 23F and 33F
PCV13–7 serotypes	1, 3, 5, 6A, 7F, 19A
PCV15–13 serotypes	22F, 33F
PCV20–15 serotypes	8, 10A, 11A, 12F, 15B
PCV20–13 serotypes	8, 10A, 11A, 12F, 15B, 22F, 33F
Non-PCV serotypes	Any serotype not contained in PCV20 (and therefore PCV15, PCV13 and PCV7)

*PCV, Pneumococcal conjugate vaccine; PPV-23, Pneumococcal polysaccharide vaccine, 23 valent.

Appendix Table 2. Demographic comparison between invasive and noninvasive pneumococcal disease*

Variable	Characteristic	Invasive disease<br>Value, n = 1,686	Noninvasive disease<br>Value, n = 2,033	p value
Age	Median [IQR]	65.9 [50.8–79.0]	66.3 [50–78.8]	0.74
Sex	Male % (no.)	50.2% (847)	48.8% (993)	0.41
	Female % (no.)	49.8% (839)	51.2% (1,040)	
Serotype Status	Serotype identified % (no.)	89.0% (1501)	0.0% (0)	<0.001
	No serotype % (no.)	11.0% (185)	100.0% (2,033)	
Smoker	Nonsmoker % (no.)	29.4% (495)	29.4% (597)	0.95
	Exsmoker % (no.)	40.3% (680)	40.8% (829)	
	Current smoker % (no.)	30.3% (511)	29.9% (607)	
CCI	Median [IQR]	4 [1–6]	4 [1–6]	0.31
Test Type	Blood culture only % (no.)	78.6% (1325)	0.0% (0)	<0.001
	UAT only % (no.)	3.0% (51)	100.0% (2,033)	
	Blood culture and UAT % (no.)	17.5% (295)	0.0% (0)	
	CSF PCR % (no.)	0.5% (8)	0.0% (0)	
	Blood PCR % (no.)	0.4% (7)	0.0% (0)	
Infection Site	Lung % no.)	84.2% (1,419)	99.2% (2,017)	<0.001
	Meningitis % (no.)	10.2% (172)	0.0% (0)	
	Septic arthritis % (no.)	2.1% (36)	0.0% (0)	
	ENT % (no.)	0.9% (15)	0.1% (2)	
	Other % (no.)	2.6% (44)	0.7% (14)	
PPV23 Vaccination	No % (no.)	57.4% (967)	57.6% (1171)	0.99
	Under 6 mo % (no.)	3.6% (60)	3.5% (72)	
	Over 6 mo % (no.)	39.1% (659)	38.8% (789)	
	Missing % (no.)	0.0% (0)	0.0% (1)	

Variable	Characteristic	Invasive disease	Noninvasive disease	p value
		Value, n = 1,686	Value, n = 2,033	
Non-Invasive Ventilation	yes % (no.)	2.3% (39)	5.1% (103)	<0.001
Intubation	yes % (no.)	14.0% (236)	11.5% (234)	0.026
Inpatient Death	yes % (no.)	15.1% (255)	12.4% (253)	0.019

*Significance determined using Fisher exact test (categorical variables) or 2 sample Kolmogorov-Smirnov test, 2 sample Wilcoxon Rank-sum test (continuous variables). Normality of distributions determined using Anderson-Darling normality test. CCI, Charlson comorbidity index; CSF, cerebrospinal fluid; ENT, ear, nose and throat; IQR, interquartile range; PCR, polymerase chain reaction; PPV23, pneumococcal polysaccharide vaccine 23-valent; UAT, urinary antigen test.

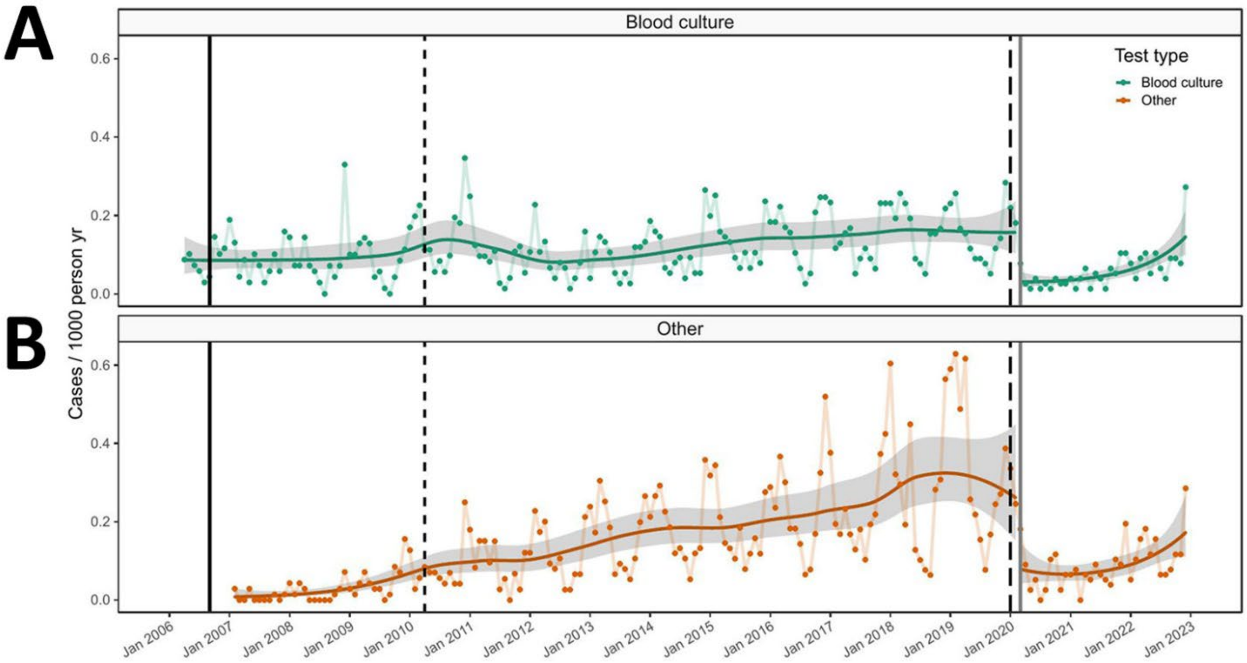

Appendix Figure 1. Test-positive pneumococcal cases in invasive and noninvasive cohorts by test method. The incidence of detected pneumococcal disease is mostly stable for blood cultures (A), and blood culture testing rate has been stable over time, but other (B) testing (largely BinaxNOW) has increased over the study period (see Figure 1, panel C) and thus there is an ascertainment bias in the case numbers in the noninvasive (i.e., Test type: Other) cohort.

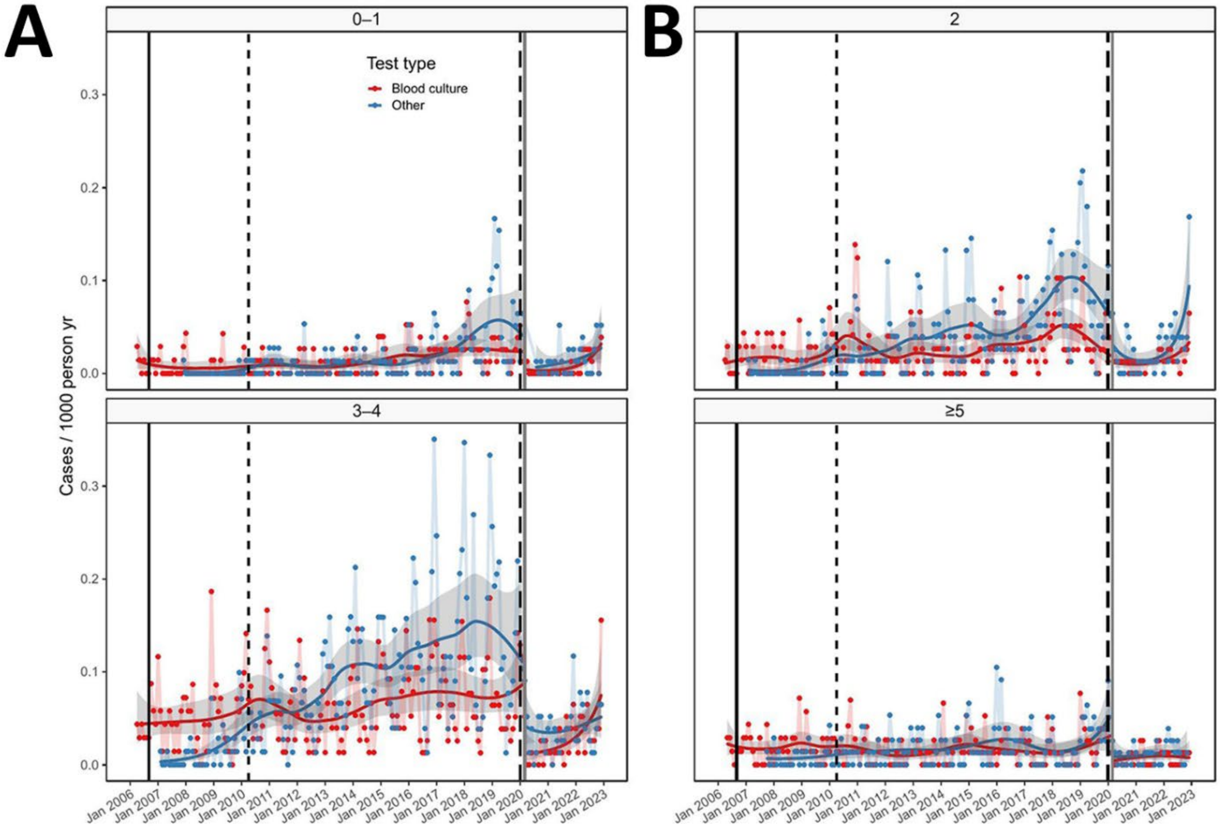

Appendix Figure 2. Test-positive pneumococcal cases in A) invasive and B) noninvasive cohorts by test method and CURB65 score Ascertainment bias in the principally noninvasive (i.e., Test type: Other) cohort is not clearly related to severity, and proportionality through time is broadly similar for different CURB65 scores. Severity analysis is focused only on invasive disease and thus is not affected by these changes in ascertainment over the study period.

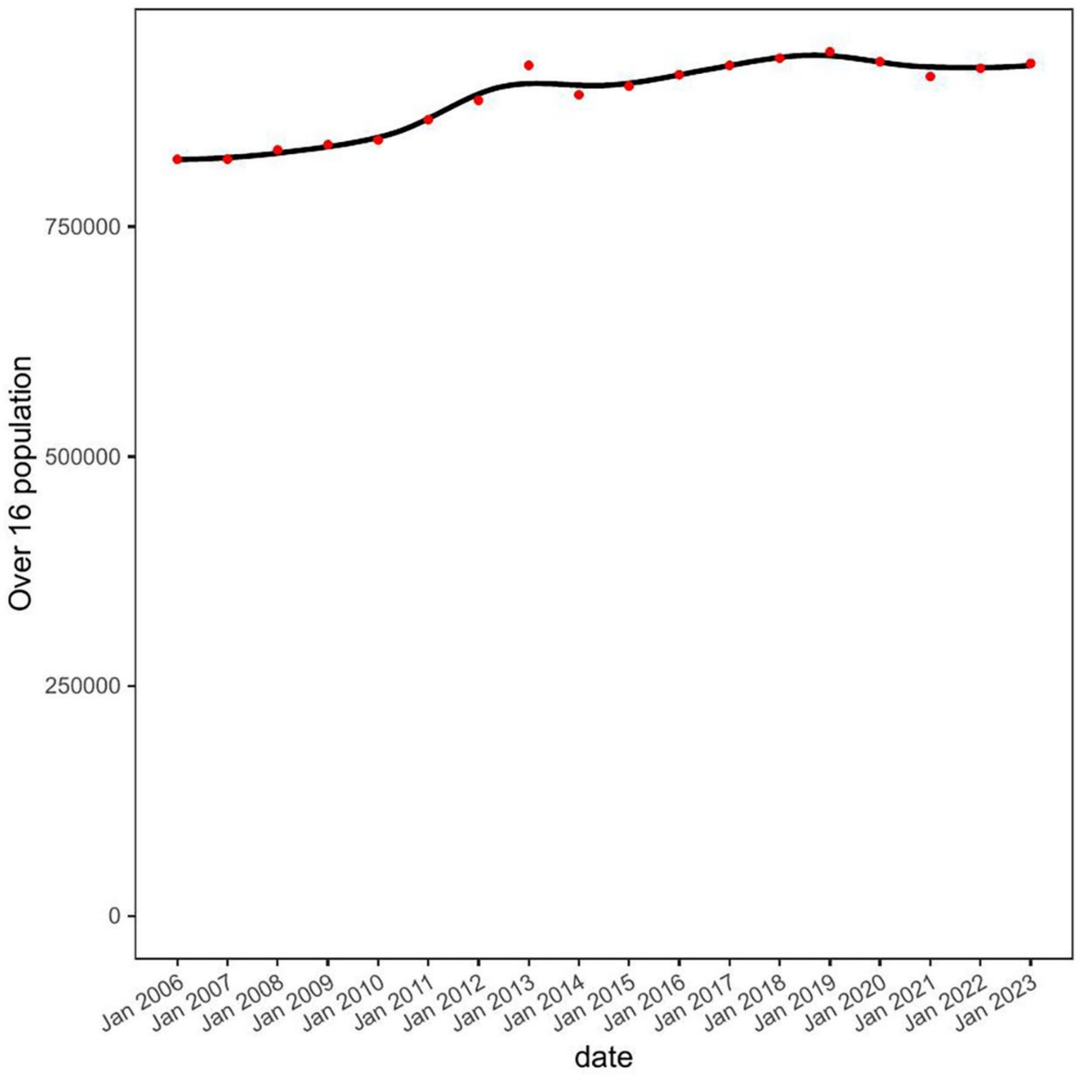

Appendix Figure 3. Population estimates in groups >16 years of age.

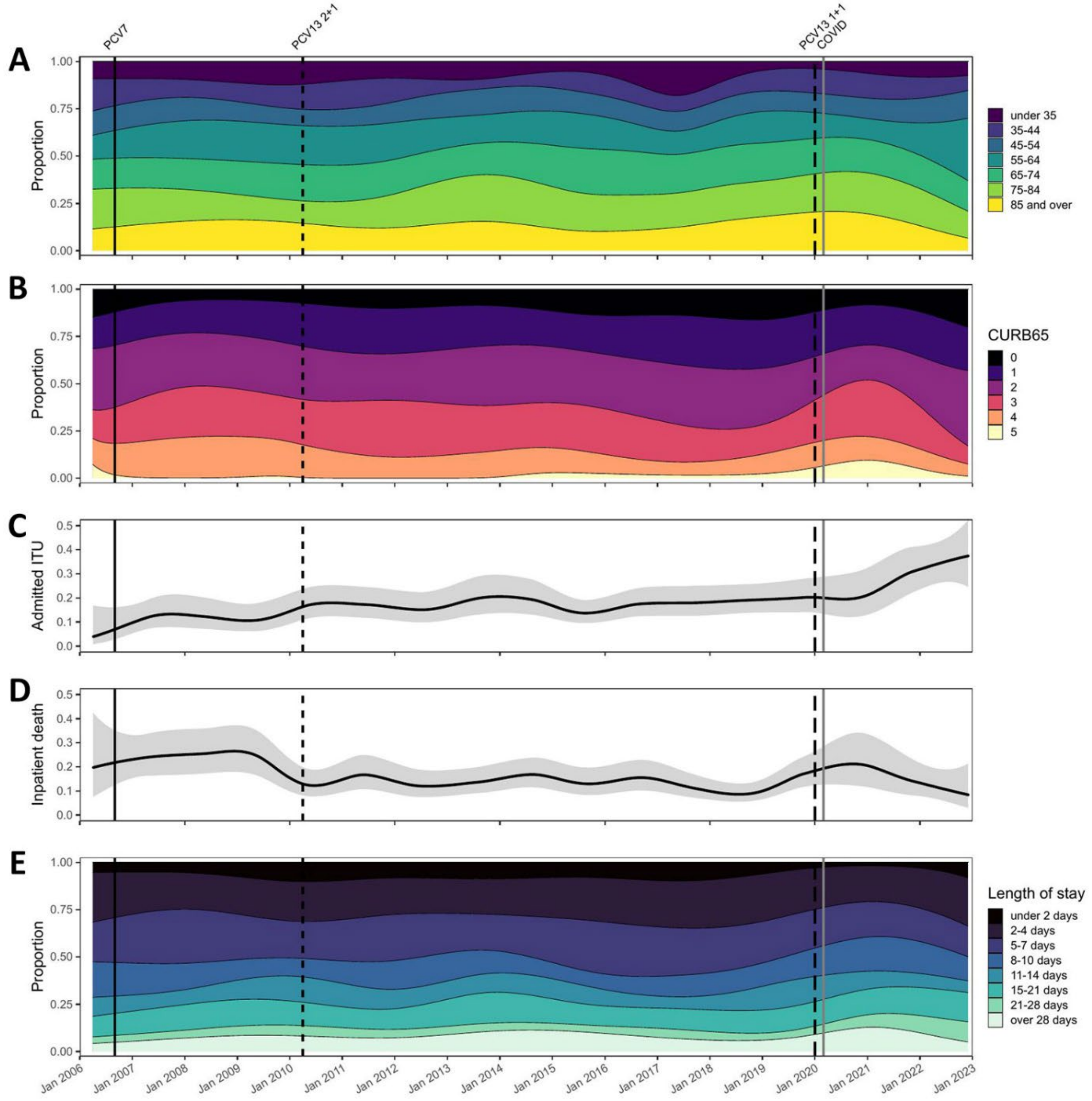

Appendix Figure 4. Severity of presentation and outcome of noninvasive pneumococcal disease over time. The percentage of noninvasive pneumococcal disease in hospitalized adults by (A) age category, (B) CURB65 score, (C) ICU admissions, (D) inpatient death and (E) hospital admission length. For panels (A), (B) and (E), categories are provided in the legend adjacent to each panel. The solid line in the multinomial time series models shown in panels (C) and (D) represents binomial time series models with 95% CIs shown as gray areas. Across all panels, the dates of PCV7 and PCV13 (2 + 1 and 1 + 1 schedule) vaccine introduction and SARS-CoV-2 emergence are shown. Because this figure relies on UAT data, the early part of the time series should be interpreted with caution because routine use was not fully established until ≈2010, and major ascertainment bias exists.
